# Identification of novel biomarkers for the prediction of subclinical coronary artery atherosclerosis in patients with rheumatoid arthritis: an exploratory analysis

**DOI:** 10.1186/s13075-023-03196-3

**Published:** 2023-10-30

**Authors:** Joan M. Bathon, Michael Centola, Xiaoqian Liu, Zhicheng Jin, Weihua Ji, Nicholas S. Knowlton, Iván Ferraz-Amaro, Qin Fu, Jon T. Giles, Mary Chester Wasko, C. Michael Stein, Jennifer E. Van Eyk

**Affiliations:** 1 Division of Rheumatology, Department of Medicine, Columbia University Irving Medical. Center/New York Presbyterian Hospital, New York, NY USA; 2 Haus Bioceuticals, Oklahoma City, OK USA; 3 Department of Pharmacy, University of Texas MD Anderson Cancer Center, Houston, TX USA; 4 Department of Pathology and Laboratory Medicine, University of Wisconsin, Madison, WI USA; 5 Mass Spectrometry Data Center Group, National Institutes of Standards and Technology, Gaithersburg, MD USA; 6 Department of Obstetrics and Gynecology, University of Auckland, Auckland, New Zealand; 7 Division of Rheumatology, Hospital Universitario de Canarias, Tenerife, Santander, Spain; 8 Cedars-Sinai Medical Center, Advanced Clinical Biosystems Research Institute, The Smidt Heart Institute, Los Angeles, CA USA; 9 Division of Rheumatology, Allegheny Health Network, Pittsburgh, Philadelphia USA; 10 Division of Rheumatology, Vanderbilt University, Nashville, TN USA

**Keywords:** Rheumatoid arthritis, Coronary artery calcification, Cardiovascular risk

## Abstract

**Background:**

Cardiovascular (CV) risk estimation calculators for the general population underperform in patients with rheumatoid arthritis (RA). The purpose of this study was to identify relevant protein biomarkers that could be added to traditional CV risk calculators to improve the capacity of coronary artery calcification (CAC) prediction in individuals with RA. In a second step, we quantify the improvement of this prediction of CAC when these circulating biomarkers are added to standard risk scores.

**Methods:**

A panel of 141 serum and plasma proteins, which represent a broad base of both CV and RA biology, were evaluated and prioritized as candidate biomarkers. Of these, 39 proteins were selected and measured by commercial ELISA or quantitative mass spectroscopy in 561 individuals with RA in whom a measure of CAC and frozen sera were available. The patients were randomly split 50:50 into a training/validation cohort. Discrimination (using area under the receiver operator characteristic curves) and re-classification (through net reclassification improvement and integrated discrimination improvement calculation) analyses were performed first in the training cohort and replicated in the validation cohort, to estimate the increase in prediction accuracy for CAC using the ACA/AHA (American College of Cardiology and the American Heart Association) score with, compared to without, addition of these circulating biomarkers.

**Results:**

The model containing ACC/AHA score *plus* cytokines (osteopontin, cartilage glycoprotein-39, cystatin C, and chemokine (C–C motif) ligand 18) and *plus* quantitative mass spectroscopy biomarkers (serpin D1, paraoxonase, and clusterin) had a statistically significant positive net reclassifications index and integrated discrimination improvement for the prediction of CAC, using ACC/AHA score without any biomarkers as the reference category. These results were confirmed in the validation cohort.

**Conclusion:**

In this exploratory analysis, the addition of several circulating CV and RA biomarkers to a standard CV risk calculator yielded significant improvements in discrimination and reclassification for the presence of CAC in individuals with RA.

**Supplementary Information:**

The online version contains supplementary material available at 10.1186/s13075-023-03196-3.

## Introduction

People with rheumatoid arthritis (RA) have higher rates of cardiovascular disease (CVD) morbidity and mortality than the general population [1], including myocardial infarctions and strokes. Traditional CV risk factors do not account entirely for the increased CVD risk in RA, and a chronically elevated level of inflammation in RA is thought to be a significant contributor to CV risk [2–4]. Indeed, CVD risk prediction models like *Framingham Risk Score* (FRS) or the *Systematic Coronary Risk Evaluation* (SCORE) algorithm, which were originally developed for use in the general population and rely heavily on traditional CVD risk factors, underestimate CV risk in patients with RA [5, 6]. A study undertaken to assess the predictive ability of four established CV risk models (SCORE, Framingham Risk Score, Reynolds Risk Score) for the 10-year risk of fatal and non-fatal CV disease in patients with RA established that these risk models generally underestimate or overestimate (QRisk II) CV risk in patients with RA [6].

In recent years, there has been an effort to build and validate novel algorithms or scores that are able to predict the occurrence of CVD specifically in RA patients. However, these new tools have had conflicting results. For example, adaptation of the SCORE algorithm by inclusion of multiple RA disease-related variables did not provide sufficient improvement in risk prediction of future CVD in RA to serve as an appropriate alternative to the original SCORE [7]. Another recently developed expanded risk score for CV events in RA also contained several RA disease-related variables [8]; however, multiple external validation studies produced variable results in its ability to improve the classification of CV risk in comparison to risk prediction scores based solely on traditional CV risk factors [9–11]. Of note, none of these new or adapted scores in RA incorporated circulating biomarkers into its predictive capacity.

A number of circulating biomarkers that reflect inflammatory, vascular, and lipid pathways involved in atherosclerosis and plaque rupture have been shown to be predictive of future CV events in the general population [12]. Indeed, several now have applications in screening, diagnosis, prognostication, prediction of disease recurrence, and therapeutic monitoring of CVD [13–15]. Combinations of biomarkers into a single multimarker may have even higher predictive capacity for CV events than single biomarkers [16–19]. However, a proteomic multimarker approach to delineate CV risk in patients with RA is lacking.

Interestingly, many CV biomarkers reflect biological pathways that are also activated in synovial inflammation in RA. In the present report, we have assessed a panel of candidate serum and plasma proteins, based on the literature, which represent a broad base of both CV and RA biology, in combined RA cohorts in whom a measure of coronary atherosclerosis, coronary artery calcification (CAC), and frozen sera were available. We selected a subclinical surrogate CV outcome, CAC, due to the availability of RA cohorts with both contemporaneous CAC and frozen sera, whereas RA cohorts with both long-term clinical CV event data and baseline frozen sera were not available at the time this study was initiated. Nonetheless, CAC is a recognized independent risk factor for CV events such as myocardial infarctions, and CAC levels are higher in RA patients compared to non-RA controls even after adjustment for conventional CV risk factors [20, 21]. The purpose of this study was to identify relevant protein biomarkers that could be added to the standard ACA/AHA score (American College of Cardiology and American Heart Association) [22] model to improve capacity for CAC prediction in individuals with RA. Our secondary aim was to quantify the improvement in the prediction of CAC with the assessment of these circulating biomarkers when they are added to the traditional CV risk factors used in a standard risk score.

## Material and methods

### Study participants and data collection

A total of 561 patients with RA from three different clinic-based cohorts (the Johns Hopkins University ESCAPE-RA cohort, the University of Pittsburgh RA cohort, and the Vanderbilt University RA cohort) were included in this study. All assessments and measurements were from baseline visits. All patients were men or women who fulfilled the American Rheumatism Association 1987 revised criteria for the classification of RA [23]. Details regarding clinic-based cohorts are described in the *Clinic-based cohorts section* of Additional file 1: Sect. 1. Each study was approved by the Institutional Review Board of the institutions of the three cohorts with all subjects providing written informed consent prior to enrollment.

### Clinical assessments

Age, gender, race/ethnicity, and current and past smoking were assessed from patient self-report. Current and past use of glucocorticoids and biologic and non-biologic disease-modifying antirheumatic drugs (DMARDs) was queried by examiner-administered questionnaires. Disease activity was measured using the Disease Activity Score in 28 joints (DAS28) with ESR or CRP, while the degree of disability was determined using the Health Assessment Questionnaire (HAQ). Metabolic syndrome was defined using the 2005 National Cholesterol Education Program Adult Treatment Panel III (ATP III) criteria. The 2008 Framingham score was calculated using age, sex, diabetes, smoking, cholesterol and HDL cholesterol levels, systolic blood pressure, and antihypertensive treatment as previously described [24]. Fasting plasma and serum samples were collected and frozen at − 80 °C until analysis. Circulating lipids, glucose, insulin, high-sensitivity CRP, and homocysteine levels were measured at each center as previously described [20, 25, 26]. Rheumatoid factor was assessed by nephelometry with seropositivity defined at or above a level of 40 units. ACC/AHA score was calculated as previously described [22]. Coronary artery calcium (CAC) quantification is explained in Additional file 1: Sect. 2.

### Candidate biomarker selection

An exhaustive literature search of established soluble proteins involved in cardiovascular biology, using broad MESH search terms including “atherosclerosis” and “cardiovascular disease” with and without restriction to RA, was performed. Initially, the Ingenuity® software (IPA®, QIAGEN Redwood City, www.qiagen.com/ingenuity) was used to mine the diverse literature on CVD and atherosclerosis. This work was supplemented with manual literature searches and with published comprehensive CVD literature reviews [27] to compile our list of candidate biomarkers. From this search, we identified 141 soluble protein-based potential candidate CV biomarkers. The evidence supporting each marker was reviewed, and a numerical ranking (1 through 4, with levels 1 and 2 having the highest strength of evidence) was assigned to each marker based on the strength of the evidence (Additional file 1: Table S1).

Of the original list of 141 candidate proteins, 63 proteins with priority levels 1 and 2 were considered for assay development based on (i) commercial availability of ELISA or (ii) if the concentration in serum would allow detection by mass spectrometry (MS). Commercially available R&D duosets were identified for 42 markers. However, 12 of these assays had poor performance characteristics and were eliminated from further consideration, leaving a total of 30 proteins analyzed by ELISA (Additional file 1: Table S1). For priority 1 and 2 proteins for which either there was no commercially available immunoassay kit or for which the kits had poor performance characteristics, quantitative MS using liquid chromatography MS/MS-based multiple reaction monitoring (MRM) method was utilized to establish a multiplex assay; however, the levels of 4 of the 12 proteins were below levels of detection of the mass spectrometer and were eliminated from further consideration, leaving a total of 8 proteins analyzed by multiplex MRM (Additional file 1: Table S1). ELISAs and optimization and targeted MRM assays for protein quantification are shown in Additional file 1: Sect. 3.

### Statistical analysis

For RA-related data, the distributions of all variables were examined. The means and standard deviations were calculated for all normally distributed continuous variables, and the medians and interquartile ranges were calculated for continuous variables that were not normally distributed. For categorical variables, counts and percentages were calculated. Logistic regression was used to establish the relation of demographics, traditional CV risk factors, analytical data (lipids, glucose, insulin, etc.), and RA-related variables with CAC higher than 100 and 300 Agatston units. Modeling of the associations of biomarkers with CAC data is shown in Additional file 1: Sect. 4.

Discrimination, re-classification, and calibration assessment. Prediction models for CAC > 100 and CAC > 300 were built using the ACC/AHA score, the ELISA and MRM proteins identified in the MV analyses, and the RA-related variables that were significantly associated with CAC. To estimate the increase in prediction accuracy between models, we used logistic regression to calculate the receiver-operator characteristic (ROC) curves and the area under the receiver-operator characteristic curves (AUC) including 95% confidence intervals and compared the AUCs to the one built with the ACC/AHA score alone. ACC/AHA score AUC was thus considered the reference and was compared with the following models: (1) ACC/AHA score plus ELISA assayed biomarkers, (2) ACC/AHA score plus MRM assayed biomarkers, and (3) ACC/AHA score plus ELISA and MRM biomarkers. A comparison of ROC curves to test the statistical significance of the difference between the areas under two dependent ROC curves (derived from the same cases) was conducted with the method of DeLong et al. [28]. Reclassification differences between models were studied through the net reclassification index (NRI) and integrated discrimination improvement (IDI) as previously described [29]. NRI and IDI range from − 2 to + 2. The NRI is the summation of the net magnitude of reclassification for those with and without the event of interest. Among those with an event, it is the proportion who are reclassified up a category minus those who are reclassified down. Among those without an event, it is the proportion of those who are reclassified down a category minus those who are reclassified up. In other words, it quantifies the degree of improved classification among those with and without events, penalized for any worsening of classification that may also occur. Risk categorization for the NRI calculation was defined using risk thresholds of < 0.5 and ≥ 0.5 for the presence of CAC. This means NRI was defined as the improvement in reclassification of having a ≥ 0.5 probability of a CAC > 100 or 300 compared to the reference category of < 0.5 probability of having CAC ≤ 100 or 300. NRI was calculated as follows: NRI = [Probability (being correctly reclassified to a higher-risk category/event) − Probability (being incorrectly reclassified to a lower-risk category/event)] – [Probability (being correctly reclassified to a lower-risk category/nonevent) − Probability (being incorrectly classified to a higher-risk category/nonevent)].

IDI was calculated as follows: IDI = (IS new − IS old) − (IP new − IP old). IS refers to the integral of sensitivity over all possible cutoff values from the (0, 1) interval and IP is the corresponding integral of “one minus specificity.” Subscript “new” refers to the model with the new markers and subscript “old” to the model without them. Since the integrals of sensitivity and “one minus specificity” over the (0, 1) interval can be seen as average sensitivity and “one minus specificity,” the IDI can be viewed as a difference between improvement in the average sensitivity and any potential increase in the average “one minus specificity.” IDI does not use cutoffs in its calculation.

Similarly, the calibration of the models was calculated using the Hosmer–Lemeshow goodness-of-fit test by grouping individuals on the basis of deciles [30, 31]. Our cohort of 561 subjects was randomly divided, 50:50 ratio, into a training sample and a validation sample. Thus, the statistical analyses were executed in the training cohort and model performance evaluated in the validation cohort. Analyses were performed using the STATA software, version 17/SE (Stata Corp., College Station, TX, USA). An alpha of 0.05 was considered statistically significant.

## Results

### Demographic, laboratory, and clinical characteristics

The baseline clinical characteristics of the patients in the three cohorts are presented in Table 1. The average age across the three cohorts was 57.6 ± 10.5 years old, 77% of the patients were women, and 89% of the patients were White race. The median CAC score was 3.12 (interquartile range 0–134.35) in the three cohorts, and 21% and 35% of the patients had a CAC score greater than the 90th and 75th percentiles, respectively. RA disease duration was 10 (interquartile range 3–20) years, 44% of the patients were receiving corticosteroid treatment, 32% were receiving anti-tumor necrosis factor (TNF) therapy, and 65% were seropositive for rheumatoid factor (Table 1). Frequencies of prior cardiovascular events were higher in the Pittsburgh and Vanderbilt cohorts, since prior events were not the exclusion criteria in these cohorts whereas they were exclusions in the Hopkins cohort. Cardiovascular comorbidities were not different between the groups except for a higher prevalence of hypertension, current smoking, and aspirin intake in the Vanderbilt cohort. There were no differences between the cohorts regarding CAC Agatston score (Table 1).

**Table 1:** Demographic and clinical data of rheumatoid arthritis patients in the three cohorts

	Total (*n* = 561)	Cohorts			
		ESCAPE (*n* = 197)	Pittsburgh (*n* = 195)	Vanderbilt (*n* = 169)	*p*
Demographics					
Age, years	57.6 ± 10.5	59.4 ± 8.7	58.8 ± 10.2	54.2 ± 11.8	** < 0.001**
Sex, female	430 (77)	118 (60)	195 (100)	117 (69)	** < 0.001**
BMI, kg/m^2^	28.5 ± 6.0	28.3 ± 5.3	27.9 ± 6.0	29.2 ± 6.8	0.133
Hip circumference, cm					
Female	106.7 ± 14.8	105.5 ± 14.6	105.9 ± 14.3	109.4 ± 15.6	0.083
Male	103.0 ± 12.3	100.6 ± 10.7	–	106.7 ± 13.6	**0.005**
Waist circumference, cm					
Female	91.9 ± 16.4	91.7 ± 15.6	91.2 ± 16.7	93.1 ± 16.8	0.640
Male	101.4 ± 14.9	101.6 ± 13.3	–	101.1 ± 17.2	0.839
Race					
White	502 (89)	169 (86)	184 (94)	149 (88)	**0.017**
Others	57 (10)	28 (14)	10 (5)	19 (11)	
Afro-American	44	18	8	18	**0.048**
Asian	10	7	2	1	0.064
Hispanic	3	3	0	0	0.063
Agatston score for CAC					
CAC Agatston units	3.12 (0–134.35)	4.69 (0–175.00)	2.75 (0–93.37)	1.85 (0–150.35)	0.798
CAC > 100	169 (30)	69 (35)	48 (25)	52 (31)	
CAC > 300	92 (16)	34 (17)	25 (13)	33 (20)	
CAC greater than 0 units	307 (55)	107 (55)	117 (60)	83 (51)	0.200
CAC greater than 10 units	248 (49)	90 (46)	86 (44)	72 (44)	0.889
CAC greater than percentile 75	195 (35)	63 (32)	75 (39)	57 (35)	0.421
CAC greater than percentile 90	113 (21)	36 (18)	39 (20)	38 (23)	0.520
Previous cardiovascular disease					
Myocardial infarction	17 (3)	0 (0)	6 (3)	11 (7)	**0.001**
Coronary artery bypass graft	8 (1)	0 (0)	1 (1)	7 (4)	0.091
Percutaneous transluminal coronary angioplasty	8 (1)	0 (0)	4 (2)	4 (2)	0.072
Stroke	9 (2)	0 (0)	1 (1)	8 (5)	**0.001**
Angina	15 (3)	0 (0)	7 (4)	8 (5)	**0.012**
Cardiac heart failure	4 (1)	0 (0)	2 (1)	2 (1)	0.331
Comorbidity					
Hypertension	235 (42)	76 (38)	71 (36)	88 (52)	**0.007**
Systolic blood pressure, mmHg	129 ± 20	128 ± 19	125 ± 19	133 ± 20	** < 0.001**
Diastolic blood pressure, mmHg	76 ± 10	76 ± 9	76 ± 10	75 ± 11	0.360
Use of antihypertensives	210 (37)	79 (40)	67 (34)	64 (38)	0.497
Dyslipidemia	119 (21)	62 (31)	57 (29)	–	0.199
On lipid lowering drugs	60 (11)	35 (18)	25 (13)	–	0.174
Diabetes	22 (4)	12 (6)	10 (5)	–	0.670
Previous smoking	291 (52)	115 (58)	96 (49)	80 (47)	0.062
Current smoking	81 (14)	23 (12)	17 (9)	41 (24)	** < 0.001**
Packs/years	0 (0–22)	7 (0–30)	0 (0–14)	0 (0–22)	**0.008**
Post menopause	249 (44)	92 (47)	157 (81)	-	** < 0.001**
Hormone replacement use	85 (15)	16 (8)	69 (35)	-	**0.047**
Metabolic syndrome	90 (16)	44 (22)	46 (24)	-	0.704
Aspirin	102 (18)	34 (17)	14 (7)	54 (32)	** < 0.001**
Analytical parameters					
Total cholesterol, mg/dL	197 ± 39	195 ± 38	208 ± 37	186 ± 39	** < 0.001**
Triglycerides, mg/dL	1116 (86–157)	–	120 (90–156)	111 (80–158)	0.151
HDL cholesterol, mg/dL	54 ± 16	49 (41–67)	61 ± 15	43 (37–54)	** < 0.001**
LDL cholesterol, mg/dL	116 ± 33	116 ± 31	120 ± 35	112 ± 33	0.100
Glucose, mg/dL	91 ± 19	89 (83–98)	88 (82–94)	87 (83–94)	0.104
Insulin, uU/ml	9. 99 ± 7.17	5.83 (3.71–9.75)	11.5 (8.60–14.20)	–	** < 0.001**
Homocysteine	10.7 ± 3.6	9.1 (7.5–10.6)	11.1 (9.6–13.7)	10.5 ± 3.4	** < 0.001**
Creatinine, mg/dL	0.8 ± 0.3	0.8 ± 0.2	0.9 ± 0.3	0.8 ± 0.2	**0.014**
CRP, mg/L	4.65 (1.75–12.50)	–	5.62 (1.97–13.35)	4.00 (1.22–13.02)	0.035
RA-associated characteristics					
Disease duration, years	10 (3–20)	9 (4–17)	13 (7–23)	3 (2–18)	** < 0.001**
Prednisone					
Ever prednisone treatment	458 (82)	147 (75)	172 (88)	139 (82)	**0.002**
Current treatment with prednisone	246 (44)	76 (39)	78 (40)	92 (54)	** < 0.001**
Current prednisone dosage, mg/day	0 (0–5)	0 (0–5)	0 (0–5)	2 (0–5)	**0.011**
Hydroxychloroquine	125 (22)	47 (24)	36 (18)	42 (25)	0.277
Methotrexate	360 (64)	125 (63)	115 (59)	120 (71)	0.056
TNF inhibitors	179 (32)	85 (43)	59 (30)	35 (21)	** < 0.001**
Anakinra	2 (0)	1 (1)	1 (1)	0 (0)	0.649
NSAIDs	313 (73)	127 (64)	135 (69)	51 (30)	** < 0.001**
COX2 inhibitors	154 (72)	47 (24)	56 (29)	51 (30)	0.356
Minutes of morning stiffness	30 (5–60)	15 (5–30)	30 (0–60)	30 (10–90)	** < 0.001**
Current biologic DMARD use	149 (27)	89 (45)	60 (31)	–	**0.003**
Any current use of non-biologic DMARDs	487 (87)	165 (84)	175 (90)	147 (87)	0.265
Joint surgery	156 (28)	55 (28)	101 (52)	–	** < 0.001**
Rheumatoid nodules	197 (35)	89 (45)	108 (55)	–	**0.012**
Global assessment of disease activity	24 (9–47)	21 (5–47)	19 (6–34)	30 (16–55)	** < 0.001**
Modified HAQ (VU cohort only)	0.500 (0.000–0.875)	–	–	0.500 (0.000–0.875)	-
DAS28 (ESCAPE and VU cohorts)	3.72 ± 1.35	3.66 ± 1.08	–	3.79 ± 1.61	0.362
Full HAQ (ESCAPE cohort only)	0.625 (0.125–1.250)	0.625 (0.125–1.250)	–	–	–
RF (> 40 units)	363 (65)	129 (65)	117 (60)	117 (69)	0.136

Data expressed as mean (± standard deviation) or median (interquartile range). Dichotomous variables are expressed as number (percentage)

Modified HAQ is only available for Vanderbilt cohort, DAS28 only for ESCAPE and Vanderbilt cohort, and full HAQ only in ESCAPE series

Significant *p* values are depicted in bold. Comparisons are performed through chi^2^, ANOVA, or Kruskall-Wallis method

*CRP* C-reactive protein, *HAQ* Health Assessment Questionnaire, *DAS28* Disease Activity Score, *HDL* high-density lipoprotein, *LDL* low-density lipoprotein, *DMARD* disease-modifying antirheumatic drug, *TNF* tumor necrosis factor, *COX-2* cyclooxygenase-2

### Demographic, co-morbidity, and RA-related data association with CAC

Age, male gender, and waist circumference were associated with both the presence of CAC higher than 100 and 300 Agatston units (Additional file 1: Table S4). Similarly, all traditional CV risk factors such as hypertension, diabetes, smoking packs/years, and use of lipid lowering drugs were associated with a higher CAC. Additionally, systolic blood pressure as a continuous variable, the use of antihypertensive drugs, previous smoking status, and the use of aspirin, were all positively associated with CAC. With regard to laboratory measures, while glucose, homocysteine, and creatinine were related to CAC > 100 or 300 Agatston units, HDL cholesterol was associated with a protective effect for both levels of CAC. Concerning RA-related data, disease duration was the only RA feature positively related to CAC. HAQ, disease activity (DAS28), and the presence of rheumatoid factor (RF) showed a trend in association with higher CAC; however, statistical significance was not reached. Regarding RA therapies, methotrexate use was associated with a lower risk for the presence of CAC higher than both 100 and 300 Agatston units, while NSAIDs were associated with a protective effect only for patients in the higher than 300 units category. The use of TNF inhibitors was not associated with CAC (Additional file 1: Table S4).

### Association of protein biomarkers with CAC: univariate and multivariable analyses

Eight ELISA markers and three MRM markers were identified as significant in the univariate analyses. The ELISA markers were cystatin C, CD40 ligand, leptin, osteopontin (OPN), osteoprotegerin (OPG), chemokine (C–C motif) ligand 18 (CCL18/MIP-4/PARC), TNF receptor 1 (TNFR1), and cartilage glycoprotein-39 (YKL40). The three MRM markers were serpin D1-NFGYTLR, paraoxonase (PON1)-IQNILTEEPK, and clusterin-IDSLLENDR. In a multivariable stepwise logistic regression limited to the eight ELISA markers, only four markers retained significance: cystatin C, MIP-4/CCL18/PARC, OPN, and YKL40. Similarly, in a separate multivariable analysis with the three MRM biomarkers, all three retained significance (data not shown).

### Discrimination, re-classification, and calibration assessment

Table 2 represents the discrimination, re-classification, and calibration assessment of the models with biomarkers versus the reference ACC/AHA score model.

**Table 2:** Discrimination, re-classification, and calibration assessment of ACC/AHA score versus models with biomarkers

	Discrimination		Calibration	Reclassification				
	AUC	*p*	H–L test	% net correct				
				Reclassification	NRI	*p*	IDI	*p*
**Training cohort (*n* = 284)**								
**CAC > 100**								
ACC/AHA	0.774 (0.701–0.846)	Ref	0.58					
ACC/AHA + cytokines	0.776 (0.696–0.856)	0.27	0.21		**0.144 (0.026–0.261)**	**0.017**	**0.085 (0.046–0.125)**	** < 0.001**
CAC < 100				0.7%				
CAC > 100				15.1%				
ACC/AHA + MRM	0.741 (0.534–0.948)	0.57	0.45		0.176 (− 0.023–0.376)	0.083	**0.128 (0.065–0.192)**	** < 0.001**
CAC < 100				0%				
CAC > 100				17.6%				
ACC/AHA + cytokines + MRM	0.840 (0.709–0.971)	0.37	0.78		**0.235 (0.005–0.467)**	**0.046**	**0.181 (0.118–0.244)**	** < 0.001**
CAC < 100				0%				
CAC > 100				23.5%				
**CAC > 300**								
ACC/AHA	0.852 (0.780–0.924)	Ref	0.19					
ACC/AHA + cytokines	0.885 (0.809–0.960)	0.24	0.85		**0.315 (0.088–0.543)**	**0.007**	**0.213 (0.132–0.295)**	** < 0.001**
CAC < 300				3.1%				
CAC > 300				34.6%				
ACC/AHA + MRM	0.849 (0.710–0.988)	0.69	0.77		0.124 (− 0.158–0.406)	0.39	0.068 (− 0.006–0.141)	0.072
CAC < 300				1.9%				
CAC > 300				14.3%				
ACC/AHA + cytokines + MRM	0.940 (0.836–0.999)	0.13	0.45		0.552 (− 0.009–1.113)	0.054	**0.399 (0.058–0.205)**	**0.002**
CAC < 300				1.9%				
CAC > 300				57.1%				
**Validation cohort (*n* = 277)**								
**CAC > 100**								
ACC/AHA	0.816 (0.757–0.875)	Ref	0.037					
ACC/AHA + cytokines	0.813 (0.745–0.882)	0.70	0.47		0.070 (− 0.034–0.175)	0.19	0.024 (− 0.008–0.056)	0.15
CAC < 100				1.5%				
CAC > 100				8.5%				
ACC/AHA + MRM	0.920 (0.821–0.999)	0.76	0.11		0.199 (− 0.056–0.454)	0.13	**0.238 (0.149–0.323)**	** < 0.001**
CAC < 100				5.1%				
CAC > 100				25.0%				
ACC/AHA + cytokines + MRM	0.941 (0.872–0.999)	0.20	0.46		**0.322 (0.0014–0.631)**	**0.041**	**0.289 (0.187–0.391)**	** < 0.001**
CAC < 100				5.3%				
CAC > 100				37.5%				
**CAC > 300**								
ACC/AHA	0.831 (0.761–0.903)	Ref	0.13					
ACC/AHA + cytokines	0.826 (0.747–0.904)	0.89	0.38		0.118 (− 0.008–0.245)	0.068	0.02740 (− 0.014–0.069)	0.19
CAC < 300				0.0%				
CAC > 300				11.1%				
ACC/AHA + MRM	0.864 (0.670–0.999)	0.64	0.12		0.125 (− 0.120–0.370)	0.32	**0.091 (0.025–0.157)**	**0.007**
CAC < 300				0.0%				
CAC > 300				12.5%				
ACC/AHA + cytokines + MRM	0.867 (0.651–0.999)	0.78	< 0.001		0.125 (− 0.120–0.370)	0.32	**0.150 (0.081–0.220)**	** < 0.001**
CAC < 300				0.0%				
CAC > 300				12.5%				

Cytokines: osteopontin, cartilage glycoprotein-39, cystatin C, and chemokine (C–C motif) ligand 18

MRM biomarkers: SerD1_NFGYTLR PON1_IQNILTEEPK Clusterin_IDSLLENDR

*p* values in AUC rows represent the comparison of every model with the first one (ACC/AHA model) that is considered the reference one

NRI and IDI are expressed as its value (95% confidence interval) and *p* value

*p* value in the H–L test express the *p* value of the Hosmer–Lemeshow chi^2^ statistic test

MRM data was only available for 140 patients of the cohorts

*ACC/AHA* American College of Cardiology and American Heart Association Score, *CAC* coronary artery calcification, *MRM* multiple reaction monitoring, *AUC* area under the curve, *NRI* net reclassification index, *IDI* integrated discrimination improvement, *H–L* Hosmer–Lemeshow chi^2^ test

Discrimination was assessed through AUC in the training cohort. ACC/AHA score, representing traditional CV risk factors, showed a highly discriminatory and statistically significant AUC for both CAC ≥ 100 and CAC ≥ 300 Agatston units categories. ACC/AHA score AUC was compared with three other models that contain combinations of ELISA biomarkers, or/and MRM biomarkers, to determine the effects of these predictor classes. For both the CAC ≥ 100 and CAC ≥ 300 categories, and in both the training and validation groups, it was observed that AUC increased as cytokines, MRM, and cytokines + MRM were sequentially added to the models. However, none of these new models yielded a significant discrimination, difference between AUCs, compared to the reference model that contains only the ACC/AHA score. Models’ calibrations (through the Hosmer–Lemeshow chi^2^ test) were found to be optimal throughout the study with some exceptions. Only the model in the validation cohort that represented the ACC/AHA + cytokines + MRM model in the CAC ≥ 300 disclosed a suboptimal calibration (*p* ≤ 0.001) (Table 2).

Net reclassification index (NRI) was found to be significant when comparing ACA/AHA score + cytokines + MRM and the reference ACA/AHA score model, in the CAC ≥ 100 Agatston units subpopulation. Remarkably, this was the case for both the training and validation groups. In this sense, applying this NRI formula that considers both those correctly reclassified and those incorrectly reclassified, a significant percentage of the RA subjects when adding cytokines + MRM to ACC/AHA score was reclassified appropriately to a high-risk probability of 50% of having a CAC ≥ 100. When NRI analysis was performed in the RA population with CAC > 300 Agatston units, the training cohort model that contained the ACC/AHA score + cytokines had a statistically significant NRI, but this was not reproduced in the validation cohort.

Remarkably, most of the IDI calculation, for both the CAC ≥ 100 and CAC ≥ 300 groups, were found to be significant when comparing the new three models (ACC/AHA score + cytokines, ACC/AHA score + MRM, and ACC/AHA score + cytokines + MRM) to the reference model (ACC/AHA score) (Table 2). This was found in the training cohort and confirmed in the validation cohort.

## Discussion

In this large sample of RA patients encompassing three individual cohorts, we constructed a predictive tool for CAC detection, a sensitive measure of atherosclerosis and predictor of future cardiovascular events [32], with several proteins previously implicated in the pathogenesis of atherosclerosis or RA or both. RA and atherosclerosis share several common pathophysiologic pathways including those involved with inflammation, thrombosis, and dyslipidemia. Previous studies of biomarkers of CV risk in RA patients have been limited to clinically accessible RA or cardiac biomarkers [33, 34]. A systematic examination of multiple biomarkers representing these various pathways in a large RA sample with a common measure of atherosclerosis has been lacking. Furthermore, many prior CV risk studies in RA have not evaluated an additive contribution to ACC/AHA score or equivalent in predicting CV events in this population.

Our study used a systematic approach to identify and measure, where reagents were available, a broad array of candidate proteins implicated in either RA or atherosclerosis or both. In multivariable analyses with adjustments for both RA characteristics and traditional CV risk factors, four of the ELISA candidate proteins were independently associated with CAC. These included cystatin C, CCL18 (MIP-4/PARC), osteopontin (OPN), and YKL40 (cartilage glycoprotein-39). Cystatin C is an extracellular inhibitor of cysteine proteases, widely recognized and utilized as a biomarker of kidney function, but also reported to be a predictor of clinical cardiovascular events independently of its relationship to renal function [18, 35, 36]. In previous studies, cystatin C levels in RA patients were positively correlated with clinical and laboratory measures of inflammation, but not independently with atherosclerosis in RA [37, 38]. CCL18 (chemokine (C–C motif) ligand 18), previously known as MIP-4 (macrophage inflammatory protein-4) or PARC (pulmonary and activation-regulated chemokine), is a small chemokine that is chemotactic for activated T cells and nonactivated lymphocytes. It has been linked to a broad array of autoimmune and allergic diseases, including RA, but is also reported to be highly expressed in atherosclerotic plaques [39]. Osteopontin (OPN; also called SPP1) is an extracellular matrix protein that mediates cell migration, adhesion, and survival in many tissues including bone. It also functions as a Th1 cytokine, playing a role in chronic inflammatory and autoimmune diseases including RA [40–42]. Moreover, OPN has also been identified as a predictor of coronary artery disease and myocardial infarction [43, 44]. YKL-40 (cartilage glycoprotein-39; chitinase 3-like 1) is a secreted glycoprotein produced by inflammatory and cancer cells and has a role in inflammation, angiogenesis, and tissue remodeling. It is a known marker of RA disease activity [45] and is one of the components of a commercially available RA disease activity multi-marker (Vectra-DA™). YKL-40 is also reported to be associated with insulin resistance, endothelial dysfunction, and atherosclerosis [46, 47].

Multi-MRM was also used to measure proteins for which ELISA was not feasible. However, due to its complexity and expense, only a subset (*n* = 140) of the combined cohort was measured. SerpinD1, paraoxonase (PON1), and clusterin were each significantly associated with CAC in multivariate modeling. SerpinD1 (heparin cofactor II) is a serine protease inhibitor that has been reported to be protective against atherosclerosis [48–50] in human and mouse studies, and levels were reported in one study to be decreased in RA synovial fluids compared to healthy controls [51]. Paraoxonase (PON1) is an enzyme component of high-density lipoprotein (HDL). By preventing the formation of atherogenic oxidized low-density lipoprotein (LDL), it is thought to reduce the risk for developing atherosclerosis [52, 53]. Moreover, higher PON1 activity was associated with lower arthritis activity in both RA patients and in a mouse model of RA, and overexpression of the human PON1 transgene in mice was associated with reduced inflammatory arthritis [54]. Clusterin is a chaperone protein that binds lipids such as high- and low-density lipoproteins and has been implicated in metabolic syndrome [55, 56]; it can also be citrullinated and is elevated in patients with early RA [55, 56].

Thus, each of these proteins was independently associated with CAC within their respective ELISA or MRM models and taken together, lay the groundwork as a potential multi-marker for cardiovascular risk in RA. However, the ACC/AHA score alone is a powerful, inexpensive, and easily derived risk factor for cardiovascular disease. Therefore, it was important to determine whether these multi-markers contributed to the measured risk from the ACC/AHA score alone. We found that discrimination through AUC (that reflects the ability of a prognostic model to correctly identify a clinical status) was not different between models but that the NRI of the final model versus the reference was found to be statically significant when CAC ≥ 100 was considered as the outcome. The AUC operating characteristic curve is a popular measure of discrimination; however, it has been criticized for its insensitivity in model comparisons in which the baseline model performs well [57]. That is, the increase in the AUC depends on the strength of the baseline model, which is true to a lesser degree for the NRI [29].

The NRI depends only on the effect size of the candidate variable and its correlation with other predictors while AUC is insensitive to change and may not increase appreciably even when a new marker is statistically significant and independently associated with risk. This appears to be the case in our study where AUC differences between the final models and the reference model were not found but in which NRI was found to be significantly different. The predictive capacity of the ACC/AHA score in the general population for clinical CV events is potent and widely demonstrated [22, 24], and improvements in risk prediction and classification by adding novel risk markers, including imaging techniques and biomarkers, have been modest but statistically significant [58]. In this large study of RA patients, the contribution of ACC/AHA score to a prediction model of CAC was confirmed to perform well; however, despite this, in our study, novel biomarkers contributed a significant incremental predictive value for the presence of CAC in RA that for the CAC ≥ 100 led to the reclassification of a significant percentage of the RA subjects.

In our study, reclassification was found to be significant for the CAC ≥ 100 outcome but not for the CAC ≥ 300. We hypothesize that the population of patients with CAC ≥ 300 may represent a group of patients in which cardiovascular risk factors have a greater burden. For this reason, the addition of new biomarkers can lead to lower reclassification percentages. However, remarkably,

IDIs were significant for all the new models and for the CAC ≥ 100 and CAC ≥ 300 subsets.

The IDI is defined as the difference of the means of the outcome probabilities, estimated by the new and old models in the patients with the outcome, minus the same difference in those without the outcome. That is, the IDI represents what the new model improves on average in terms of the prediction of more true events, discounting what worsens due to the prediction of false events.

Our work has several strengths. Our cohort of RA patients is the largest with an objective measure of subclinical atherosclerosis, and our approach included, to our knowledge, the most comprehensive, systematic exam of potential protein candidates spanning multiple CV/RA pathways. However, we acknowledge some limitations. First, not all candidate proteins could be measured due to the lack of availability of test kits, or quantities below limits of detection. Second, the gold standard outcome for CV risk prediction is clinical CV events; however, RA cohorts large enough to identify adequate numbers of CV events and have available sera are not generally available. However, CAC is a suitable surrogate outcome as it is an excellent predictor of future CV events and has additive value on top of the ACC/AHA score; moreover, it is accessible, easily measured, and a linear variable, thus easier to use for biomarker evaluations. Moreover, CAC independently predicts CV events in patients with RA [59]. We also acknowledge that the contribution of the individual new biomarkers to CV prediction will require further study. While ELISA measurements are commonly used in clinical practice, MRM measurements are not. The cost of assessing these markers in practice is also unknown. For this reason, it will be necessary to study in the future whether measurement of these markers is cost-effective when implemented in clinical practice to further stratify CV risk in patients with RA.

In conclusion, in this exploratory study, the addition of several circulating biomarkers, related to both CV and RA biology, to standard CV risk calculators yielded significant improvements in discrimination and reclassification for the presence of CAC in RA patients. Our findings emphasize the importance that certain biomarkers, specifically related to the pathophysiology of RA, may have in disease-related CV disease. The identification of these biomarkers will permit a better understanding of the disease and may more correctly identify those patients with higher CV risk.

### Supplementary Information

**Additional file 1.** Section 1. Clinic-based cohorts. Section 2. Coronary Artery Calcium (CAC) quantification. Section 3. ELISAs and optimization and targeted MRM Assays for protein quantification. Section 4. Modeling of the Associations of Biomarkers with CAC Data Processing. Table S1. Potential and Final Biomarkers Selected for Assay. Table S2. Eight Protein MRM Multiplex Assay. Table S3. Characterization of MRM Multiplex Assay in the Quality Control Material. Table S4. Univariable association of demographic, co-morbidity and RA related data with CAC. 

## Data Availability

The data sets used and/or analyzed in the present study are available from the corresponding author upon request.

## Abbreviations

CRP: C-reactive protein
HAQ: Health Assessment Questionnaire
DAS28: Disease Activity Score
HDL: High-density lipoprotein
LDL: Low-density lipoprotein
DMARD: Disease-modifying antirheumatic drug
TNF: Tumor necrosis factor
COX-2: Cyclooxygenase-2
AUC: Area under the curve
NRI: Net reclassification index
IDI: Integrated discrimination improvement
H-L: Hosmer-Lemeshow chi^2^ test
