# Supplementary material for: Identification of novel biomarkers for the prediction of subclinical coronary artery atherosclerosis in patients with rheumatoid arthritis: an exploratory analysis
Source: Arthritis Res Ther. 2023 Oct 30;25:213. doi: 10.1186/s13075-023-03196-3 (PMC10614317; doi:10.1186/s13075-023-03196-3)
Supplement: Supplementary file 1 — Additional file 1. Section 1. Clinic-based cohorts. Section 2. Coronary Artery Calcium (CAC) quantification. Section 3. ELISAs and optimization and targeted MRM Assays for protein quantification. Section 4. Modeling of the Associations of Biomarkers with CAC Data Processing. Table S1. Potential and Final Biomarkers Selected for Assay. Table S2. Eight Protein MRM Multiplex Assay. Table S3. Characterization of MRM Multiplex Assay in the Quality Control Material. Table S4. Univariable association of demographic, co-morbidity and RA related data with CAC. [file 13075_2023_3196_MOESM1_ESM.docx]

***SUPPLEMENTAL MATERIAL***

***Section 1. Clinic-based cohorts***

The Johns Hopkins ESCAPE-RA cohort (Evaluation of Subclinical Cardiovascular disease And Predictors of Events in Rheumatoid Arthritis study (Principal Investigator [PI], J Bathon) has been described in detail previously [1] and was initiated to study the prevalence, progression, and risk factors for subclinical cardiovascular disease in RA. A total of 197 patients aged 45 to 80 years old with a disease duration >6 months, were recruited from the Johns Hopkins Arthritis Center. The Pittsburgh cohort (PI, MC Wasko) consisted of 195 patients recruited from the University of Pittsburgh Medical Center Arthritis Network outpatient practices. All patients in this cohort were women, 16 years old or older, with disease duration ≥ 2 years. In the Vanderbilt cohort (PI, CM Stein), patients were a minimum age of 18 years old and there were two groups of patients: duration of disease of <5 years (early RA) or >10 years (established RA). This cohort of 169 patients was drawn from a registry of patients with early RA, were referred by local rheumatologists, or were recruited by advertisements. Details of the Pittsburgh and Vanderbilt cohorts have also been previously reported [2,3].

***Section 2. Coronary Artery Calcium (CAC) quantification***

All subjects in the three cohorts underwent cardiac computerized tomography at their respective institutions using methodologies described previously [1–4]. Coronary artery atherosclerosis was assessed by quantifying the amount of CAC using the Agatston method [5]. In addition to the Agatston score as a continuous variable, CAC was also categorized as greater than 100 and greater than 300 Agatston units [6]. Because of its high correlation with incident CV events, CAC is well accepted as validated surrogate outcome for clinical cardiovascular disease [7]. CAC has also been used as an outcome measure in previous biomarker studies [8].

***Section 3. ELISAs and optimization and targeted MRM Assays for protein quantification***

*ELISAs and Optimization*

All ELISA assays were run in 96-well plates with 8 point standard curves (including blank). ELISA based assays were evaluated using a BioTek ELx800 plate reader (BioTek Inc, Winooski, VT) and TERIS software with 4 parameter logistic curve fitting and 1/y weighting and Meso Scale Discovery (MSD) assays were evaluated using a Sector Imager 6000 device (Meso Scale Discovery, Gaithersburg, MD) using MSD Discovery Workbench software with 4 parameter logistic curve fitting and 1/y2 weighting. Sera from 20 ESCAPE RA patients were used to optimize assay sensitivity and dynamic range, to best map RA patient protein measurements onto the linear portion of the standard curve, and to minimize serum volume requirements.

*Targeted MRM Assays for protein quantification.*

As with the ELISAs, the multiplex MRM assay was initially performed on a small set of RA patient sera (n=20) to assess analyte measurability and to develop multiplex profiling capabilities. The final multiplex MRM assay was performed on a subset of the entire cohort (n=140). Recombinant proteins (Sigma) were digested as outlined below and two to three unique peptides from each protein were selected for quantification based on the tryptic peptide reproducibility, sensitivity (LOD) and extent of linearity in plasma. The final MRM multiplex assay was comprised of 8 proteins (20 peptides and 40 transitions) and beta-galactosidese (2 peptides and 4 transitions) (**Supplemental Table 2**).

Plasma samples were denatured, reduced, alkylated (MMTS (200 mM) and digested either manually for Hopkins cohort [9,10]) or in a 96-well plate formate on aBiomek NX^P^ Span-8 Laboratory Automation Workstation (Beckman Coulter), according to optimized method outlined by Fu *et. al* [11]*,* for the Pittsburgh and Vanderbilt cohorts. Beta-galactosidase (Sigma-Aldrich) was added prior to denaturation and was used as a sample processing control. Synthesized stable isotope-labeled peptides (Cambridge Isotope Labs) were produced for the eight proteins and β-Gal, added after trypsin digestion, and served as internal standards.

Digested peptides were analyzed by liquid chromatrography/MS/MS on a UFLCXR HPLC (Shimadzu Prominence) with a Xbridge BEH30 C18 column (2.1mmx100mm, 3.5µm, Waters) using a QTRAP 6500 system (SCIEX). Analyst 1.6.1 Software was used for the operating system. Each patient sample was run in triplicate. Each block was composed of 40 samples. Three quality control samples were run for every block. A 10 point standard curve of the heavy isotopic labelled peptides was run at the begining. A 3 point standard curve was run every 10 samples and at the end of each batch (see **Supplementary Table 3** for %CV). All data were processed by MultiQuant 2.1 Software. All peptides for each protein were quantified.

***Section 4. Modeling of the Associations of Biomarkers with CAC Data Processing***

MRM data were imported as quantitative values from the instrument. ELISA data were fit to a 4 parameter logistic standard curve for quantification. The resulting values were logarithm base 2 transformed before being used in subsequent analysis. All analyses was performed with the R Statistical Language.

Univariable Analyses. CAC was analyzed in the combined Hopkins, Pittsburgh and Vanderbilt cohorts as a binary variable at two CAC score cut-off points, 100 and 300. Separate analyses for the two cutoffs were performed in which the data were modeled one biomarker at a time through bootstrapped logistic regression models.

Multivariable Analyses. All univariable markers identified to be statistically significantly associated with CAC were re-examined in a multivariable context (AUC) with all significant terms added to a global model. Terms were removed one at a time to determine if they had any statistical effect on the AUC - that is, if there is a statistical decrease in AUC. If no effect was observed they were retained in the overall model. This process was repeated until the number of terms in the model was stable. Due to the smaller number of patient sera assayed by MRM (n=140), the iterative selection process was applied separately on the subset of samples that were assayed for MRM markers. When more than one peptide within a given protein was significantly associated with CAC score, the peptide with the smallest p-value was retained.

**References**

1. Giles JT, Szklo M, Post W, Petri M, Blumenthal RS, Lam G, et al. Coronary arterial calcification in rheumatoid arthritis: comparison with the Multi-Ethnic Study of Atherosclerosis. Arthritis Res Ther. 2009;11.

2. Chung CP, Oeser A, Raggi P, Gebretsadik T, Shintani AK, Sokka T, et al. Increased coronary-artery atherosclerosis in rheumatoid arthritis: relationship to disease duration and cardiovascular risk factors. Arthritis Rheum. 2005;52:3045–53.

3. Kao AH, Krishnaswami S, Cunningham A, Edmundowicz D, Morel PA, Kuller LH, et al. Subclinical coronary artery calcification and relationship to disease duration in women with rheumatoid arthritis. Journal of Rheumatology. 2008;35:61–9.

4. Carr JJ, Nelson JC, Wong ND, McNitt-Gray M, Arad Y, Jacobs DR, et al. Calcified coronary artery plaque measurement with cardiac CT in population-based studies: Standardized protocol of Multi-Ethnic Study of Atherosclerosis (MESA) and Coronary Artery Risk Development in Young Adults (CARDIA) study. Radiology. Radiology; 2005. p. 35–43.

5. Nelson JC, Kronmal RA, Carr JJ, McMitt-Gray MF, Wong ND, Loria CM, et al. Measuring coronary calcium on CT images adjusted for attenuation differences. Radiology. 2005;235:403–14.

6. Goff DC, Lloyd-Jones DM, Bennett G, Coady S, D’Agostino RB, Gibbons R, et al. 2013 ACC/AHA guideline on the assessment of cardiovascular risk: A report of the American college of cardiology/American heart association task force on practice guidelines. Circulation. Circulation; 2014.

7. Budoff MJ, Achenbach S, Blumenthal RS, Carr JJ, Goldin JG, Greenland P, et al. Assessment of coronary artery disease by cardiac computed tomography: a scientific statement from the American Heart Association Committee on Cardiovascular Imaging and Intervention, Council on Cardiovascular Radiology and Intervention, and Committee on Cardiac Imaging, Council on Clinical Cardiology. Circulation. 2006;114:1761–91.

8. Möhlenkamp S, Lehmann N, Moebus S, Schmermund A, Dragano N, Stang A, et al. Quantification of coronary atherosclerosis and inflammation to predict coronary events and all-cause mortality. J Am Coll Cardiol. 2011;57:1455–64.

9. Jin Z, Fu Z, Yang J, Troncosco J, Everett AD, Van Eyk JE. Identification and characterization of citrulline-modified brain proteins by combining HCD and CID fragmentation. Proteomics. 2013;13:2682–91.

10. Fu Q, Chen Z, Zhang S, Parker SJ, Fu Z, Tin A, et al. Multiple and Selective Reaction Monitoring Using Triple Quadrupole Mass Spectrometer: Preclinical Large Cohort Analysis. Methods Mol Biol. 2016;1410:249–64.

11. Fu Q, Kowalski MP, Mastali M, Parker SJ, Sobhani K, Van Den Broek I, et al. Highly Reproducible Automated Proteomics Sample Preparation Workflow for Quantitative Mass Spectrometry. J Proteome Res. 2018;17:420–8.

| **Supplementary Table 1. Potential and Final Biomarkers Selected for Assay** | | | | |
| --- | --- | --- | --- | --- |
| **Potential ELISA markers** | **Final ELISA** | **Potential MRM markers** | **Final MRM** |  |
| **(n=42)** | **markers** | **(n=13)** | **markers (n=8)** |  |
|  | **(n=30)** |  |  |  |
| Adiponectin | Adiponectin | Apolipoprotein A1 | Apolipoprotein A1 |  |
| Angiopoietin-2 |  | Apolipoprotein B | Apolipoprotein B |  |
| CCL13/MCP-4 |  | Clusterin | Clusterin |  |
| CCL7/MCP-3 |  | Cystatin C | Cystatin C |  |
| CCL18/MIP-4/  PARC  CD40 ligand | CCL18/MIP-4/  PARC  CD40 ligand | Endothelial lipase |  |  |
| CRP | CRP | Osteocalcin |  |  |
| Cystatin C | Cystatin C | Paraoxonase | Paraoxonase |  |
| E-Selectin | E-selectin | Periostin |  |  |
| EGF | EGF | Phospholipase A2 |  |  |
| IGFBP1 | IGBP-1 | Phospholipid transfer protein | Phospholipid transfer protein |  |
| IL-18 |  | Serpin D1 (heparin cofactor 2) | Serpin D1 (heparin cofactor 2) |  |
| IL-1 beta |  | Tissue factor pathway inhibitor |  |  |
| IL-6 | IL-6 | Von Willebrand Factor | vonWillebrand Factor |  |
| IL-6R | IL-6R |  |  |  |
| IP-10 | IP-10 |  |  |  |
| L-Selectin | L-Selectin |  |  |  |
| Leptin | Leptin |  |  |  |
| Lipocalin 2 | Lipocalin 2 |  |  |  |
| M-CSF |  |  |  |  |
| MMP-1 | MMP-1  MMP-3 |  |  |  |
| MMP-3 |  |  |  |  |
| MMP-7 |  |  |  |  |
| MMP-9 | MMP-9 |  |  |  |
| Osteopontin | Osteopontin |  |  |  |
| Osteoprotegerin | Osteoprotegerin |  |  |  |
| P-Selectin | P-Selectin |  |  |  |
| PCSK9 |  |  |  |  |
| PDGF-BB |  |  |  |  |
| Placental growth factor |  |  |  |  |
| RANKL |  |  |  |  |
| Resistin | Resistin |  |  |  |
| TGF-beta1 |  |  |  |  |
| Thrombomodulin | Thrombomodulin |  |  |  |
| Thrombospondin-1 | Thrombospondin-1 |  |  |  |
| Thrombospondin-2 | Thrombospondin-2 |  |  |  |
| TIMP-1 | TIMP-1 |  |  |  |
| TIMP-3 | TIMP-3 |  |  |  |
| TNFR1 | TNFR1 |  |  |  |
| VCAM-1 | VCAM-1 |  |  |  |
| VEGFR2/sKDR | VEGFR2/sKDR |  |  |  |
| YKL-40 | YKL-40 |  |  |  |

**Supplementary Table 2. Eight Protein MRM Multiplex Assay**

| MRM Protein | Peptides | Transitions |
| --- | --- | --- |
| \| Apolipoprotein A1 \| \| --- \| \|  \| | ATEHLSTLSEK  DLATVYVDVLK  LLDNWDSVTSTFSK | 608.3/664.4  618.3/936.9  806.9/670.5 |
| \| \| ApolApolipoprotein B100 \| \| --- \| \|  \| \| \| --- \| --- \| --- \| | FPEVDVLTK  GFEPTLEALFGK | 524.2/450.9  654.8/488.3 |
| Beta-galactosidase (β-gal), control | IDPNAWVER | 550.3/436.1 |
| \| Clusterin \| \| --- \| \|  \| | SGSGLVGR  IDSLLENDR | 366.7/588.3  537.8/646.3 |
| Cystatin C (CysC) | ALDFAVGEYNK  ALQVVR | 613.8/1042.5  343.2/501.3 |
| \| Paraoxonase (PON 1) \| \| --- \| | IQNILTEEPK  STVELFK  SFNPNSPGK | 592.8/242.1  412.2/536.3  474.2/235.1 |
| \| Phospholipid transfer protein (PLTP) \| \| --- \| | AVEPQLQEEER  GAFFPLTER | 664.3/514.8  519.3/615.3 |
| \| Serpin D1 \| \| --- \| \|  \| | IAIDLFK  TLEAQLTPR  NFGYTLR | 410.2/706.4  514.8/814.4  435.7/609.3 |
| \| Von Willebrand Factor \| \| --- \| \| (VWF) \| | VTVFPIGIGDR  ILAGPAGDSNVVK  TNTGLALR | 587.3/727.4  621.2/1014.5  423.2/630.4 |

**Supplementary Table 3. Characterization of MRM Multiplex Assay in the Quality Control Material.**

| **Protein** | **Peptide** | **QC level** | **Mean (nmol/ml)** | **Standard deviation** | **CV (%)** |
| --- | --- | --- | --- | --- | --- |
| Clusterin | SGSGLVGR | Low | 10.27 | 0.37 | 3.63 |
|  |  | Medium | 25.32 | 1.46 | 5.75 |
|  |  | High | 67.33 | 3.17 | 4.71 |
| Clusterin | IDSLLENDR | Low | 13.07 | 0.4 | 3.06 |
|  |  | Medium | 32.31 | 1.21 | 3.74 |
|  |  | High | 86.11 | 2.4 | 2.79 |
| Paraoxonase | IQNILTEEPK | Low | 7.44 | 0.22 | 2.93 |
|  |  | Medium | 17.86 | 0.58 | 3.25 |
|  |  | High | 47.27 | 1.14 | 2.4 |
| Paraoxonase | STVELFK | Low | 7.26 | 0.26 | 3.65 |
|  |  | Medium | 17.38 | 0.72 | 4.16 |
|  |  | High | 45.31 | 1.45 | 3.2 |
| Paraoxonase | SFNPNSPGK | Low | 4.8 | 0.1 | 2.15 |
|  |  | Medium | 11.96 | 0.15 | 1.26 |
|  |  | High | 31.14 | 0.63 | 2.01 |
| Serpin D1 | IAIDLFK | Low | 12.5 | 0.19 | 1.55 |
|  |  | Medium | 26.37 | 0.23 | 0.87 |
|  |  | High | 64.49 | 0.57 | 0.88 |
| Serpin D1 | NFGYTLR | Low | 10.12 | 0.18 | 1.8 |
|  |  | Medium | 24.71 | 0.26 | 1.04 |
|  |  | High | 64.41 | 0.39 | 0.6 |
| Serpin D1 | TLEAQLTPR | Low | 13.54 | 0.22 | 1.62 |
|  |  | Medium | 33.89 | 0.41 | 1.22 |
|  |  | High | 87.6 | 0.94 | 1.08 |
| Apolipoprotein B | FPEVDVLTK | Low | 11.14 | 0.08 | 0.73 |
|  |  | Medium | 26.65 | 0.26 | 0.96 |
|  |  | High | 68.37 | 0.91 | 1.33 |

| **~~Supplementary Table 4. Demographic and clinical data of rheumatoid arthritis patients in the three cohorts.~~** | | | | | | | | | | | |  | |  | | |  | |  |  |  |  |  |  |  |  |
| --- | --- | --- | --- | --- | --- | --- | --- | --- | --- | --- | --- | --- | --- | --- | --- | --- | --- | --- | --- | --- | --- | --- | --- | --- | --- | --- |
|  |  | | | |  | |  | ~~Total (n=561)~~ |  | ~~Cohorts~~ | | | | | | | | |  |  |  |  |  |  |  |  |
|  |  | | | |  | |  |  |  | *~~ESCAPE (n=197)~~* | *~~Pittsburgh (n=195)~~* | | *~~Vanderbilt (n=169)~~* | | |  | | |  |  |  |  |  |  |  |  |
| ~~Demographics~~ | | | | | | |  |  |  |  |  | |  | | | *~~p~~* | | |  |  |  |  |  |  |  |  |
|  | ~~Age, years~~ | | | | | |  | ~~57.6 ± 10.5~~ |  | ~~59.4 ± 8.7~~ | ~~58.8 ± 10.2~~ | | ~~54.2 ± 11.8~~ | | | **~~<0.001~~** | | |  |  |  |  |  |  |  |  |
|  | ~~Sex, female~~ | | | | | | | ~~430 (77)~~ |  | ~~118 (60)~~ | ~~195 (100)~~ | | ~~117 (69)~~ | | | **~~<0.001~~** | | |  |  |  |  |  |  |  |  |
|  | ~~BMI, kg/m2~~ | | | | | | | ~~28.5 ± 6.0~~ |  | ~~28.3 ± 5.3~~ | ~~27.9 ± 6.0~~ | | ~~29.2 ± 6.8~~ | | | ~~0.133~~ | | |  |  |  |  |  |  |  |  |
|  | ~~Hip circumference, cm~~ | | | | | | |  |  |  |  | |  | | |  | | |  |  |  |  |  |  |  |  |
|  |  | | ~~Female~~ | | | | | ~~106.7 ± 14.8~~ |  | ~~105.5 ± 14.6~~ | ~~105.9 ± 14.3~~ | | ~~109.4 ± 15.6~~ | | | ~~0.083~~ | | |  |  |  |  |  |  |  |  |
|  |  | | ~~Male~~ | | |  | | ~~103.0 ± 12.3~~ |  | ~~100.6 ± 10.7~~ | ~~-~~ | | ~~106.7 ± 13.6~~ | | | **~~0.005~~** | | |  |  |  |  |  |  |  |  |
|  | ~~Waist circumference, cm~~ | | | | | | |  |  |  |  | |  | | |  | | |  |  |  |  |  |  |  |  |
|  |  | | ~~Female~~ | | | | | ~~91.9 ± 16.4~~ |  | ~~91.7 ± 15.6~~ | ~~91.2 ± 16.7~~ | | ~~93.1 ± 16.8~~ | | | ~~0.640~~ | | |  |  |  |  |  |  |  |  |
|  |  | | ~~Male~~ | | |  | | ~~101.4 ± 14.9~~ |  | ~~101.6 ± 13.3~~ | ~~-~~ | | ~~101.1 ± 17.2~~ | | | ~~0.839~~ | | |  |  |  |  |  |  |  |  |
|  | ~~Race~~ | | | |  | |  |  |  |  |  | |  | | |  | | |  |  |  |  |  |  |  |  |
|  |  | | ~~White~~ | | | | | ~~502 (89)~~ |  | ~~169 (86)~~ | ~~184 (94)~~ | | ~~149 (88)~~ | | | **~~0.017~~** | | |  |  |  |  |  |  |  |  |
|  |  | | ~~Others~~ | | | | | ~~57 (10)~~ |  | ~~28 (14)~~ | ~~10 (5)~~ | | ~~19 (11)~~ | | |  | | |  |  |  |  |  |  |  |  |
|  |  |  | | ~~Afro-American~~ | | | | ~~44~~ |  | ~~18~~ | ~~8~~ | | ~~18~~ | | | **~~0.048~~** | | |  |  |  |  |  |  |  |  |
|  |  |  | | ~~Asian~~ | | | | ~~10~~ |  | ~~7~~ | ~~2~~ | | ~~1~~ | | | ~~0.064~~ | | |  |  |  |  |  |  |  |  |
|  |  |  | | ~~Hispanic~~ | | | | ~~3~~ |  | ~~3~~ | ~~0~~ | | ~~0~~ | | | ~~0.063~~ | | |  |  |  |  |  |  |  |  |
| ~~Agatston score for CAC~~ | | | | | | | |  |  |  |  | |  | | |  | | |  |  |  |  |  |  |  |  |
|  | ~~CAC Agatston units~~ | | | | | | | ~~3.12 (0-134.35)~~ |  | ~~4.69 (0-175.00)~~ | ~~2.75 (0-93.37)~~ | | ~~1.85 (0-150.35)~~ | | | ~~0.798~~ | | |  |  |  |  |  |  |  |  |
|  | ~~CAC > 100~~ | | | | | | | ~~169 (30)~~ |  | ~~69 (35)~~ | ~~48 (25)~~ | | ~~52 (31)~~ | | |  | | |  |  |  |  |  |  |  |  |
|  | ~~CAC > 300~~ | | | | | | | ~~92 (16)~~ |  | ~~34 (17)~~ | ~~25 (13)~~ | | ~~33 (20)~~ | | |  | | |  |  |  |  |  |  |  |  |
|  | ~~CAC greater than 0 units~~ | | | | | | | ~~307 (55)~~ |  | ~~107 (55)~~ | ~~117 (60)~~ | | ~~83 (51)~~ | | | ~~0.200~~ | | |  |  |  |  |  |  |  |  |
|  | ~~CAC greater than 10 units~~ | | | | | | | ~~248 (49)~~ |  | ~~90 (46)~~ | ~~86 (44)~~ | | ~~72 (44)~~ | | | ~~0.889~~ | | |  |  |  |  |  |  |  |  |
|  | ~~CAC greater than percentile 75~~ | | | | | | | ~~195 (35)~~ |  | ~~63 (32)~~ | ~~75 (39)~~ | | ~~57 (35)~~ | | | ~~0.421~~ | | |  |  |  |  |  |  |  |  |
|  | ~~CAC greater than percentile 90~~ | | | | | | | ~~113 (21)~~ |  | ~~36 (18)~~ | ~~39 (20)~~ | | ~~38 (23)~~ | | | ~~0.520~~ | | |  |  |  |  |  |  |  |  |
| ~~Previous cardiovascular disease~~ | | | | | | | |  |  |  |  | |  | | |  | | |  |  |  |  |  |  |  |  |
|  | ~~Myocardial infarction~~ | | | | | | | ~~17 (3)~~ |  | ~~0 (0)~~ | ~~6 (3)~~ | | ~~11 (7)~~ | | | **~~0.001~~** | | |  |  |  |  |  |  |  |  |
|  | ~~Coronary artery bypass graft~~ | | | | | | | ~~8 (1)~~ |  | ~~0 (0)~~ | ~~1 (1)~~ | | ~~7 (4)~~ | | | ~~0.091~~ | | |  |  |  |  |  |  |  |  |
|  | ~~Percutaneous transluminal coronary angioplasty~~ | | | | | | | ~~8 (1)~~ |  | ~~0 (0)~~ | ~~4 (2)~~ | | ~~4 (2)~~ | | | ~~0.072~~ | | |  |  |  |  |  |  |  |  |
|  | ~~Stroke~~ | | | | | |  | ~~9 (2)~~ |  | ~~0 (0)~~ | ~~1 (1)~~ | | ~~8 (5)~~ | | | **~~0.001~~** | | |  |  |  |  |  |  |  |  |
|  | ~~Angina~~ | | | | | |  | ~~15 (3)~~ |  | ~~0 (0)~~ | ~~7 (4)~~ | | ~~8 (5)~~ | | | **~~0.012~~** | | |  |  |  |  |  |  |  |  |
|  | ~~Cardiac heart failure~~ | | | | | | | ~~4 (1)~~ |  | ~~0 (0)~~ | ~~2 (1)~~ | | ~~2 (1)~~ | | | ~~0.331~~ | | |  |  |  |  |  |  |  |  |
| ~~Comorbidity~~ | | | | | | |  |  |  |  |  | |  | | |  | | |  |  |  |  |  |  |  |  |
|  | ~~Hypertension~~ | | | | | | | ~~235 (42)~~ |  | ~~76 (38)~~ | ~~71 (36)~~ | | ~~88 (52)~~ | | | **~~0.007~~** | | |  |  |  |  |  |  |  |  |
|  | ~~Systolic blood pressure, mmHg~~ | | | | | | | ~~129 ± 20~~ |  | ~~128 ± 19~~ | ~~125 ± 19~~ | | ~~133 ± 20~~ | | | **~~<0.001~~** | | |  |  |  |  |  |  |  |  |
|  | ~~Diastolic blood pressure, mmHg~~ | | | | | | | ~~76 ± 10~~ |  | ~~76 ± 9~~ | ~~76 ± 10~~ | | ~~75 ± 11~~ | | | ~~0.360~~ | | |  |  |  |  |  |  |  |  |
|  | ~~Use of antihypertensives~~ | | | | | | | ~~210 (37)~~ |  | ~~79 (40)~~ | ~~67 (34)~~ | | ~~64 (38)~~ | | | ~~0.497~~ | | |  |  |  |  |  |  |  |  |
|  | ~~Dyslipidemia~~ | | | | | |  | ~~119 (21)~~ |  | ~~62 (31)~~ | ~~57 (29)~~ | | ~~-~~ | | | ~~0.199~~ | | |  |  |  |  |  |  |  |  |
|  |  | | ~~On lipid lowering drugs~~ | | | | | ~~60 (11)~~ |  | ~~35 (18)~~ | ~~25 (13)~~ | | ~~-~~ | | | ~~0.174~~ | | |  |  |  |  |  |  |  |  |
|  | ~~Diabetes~~ | | | | | |  | ~~22 (4)~~ |  | ~~12 (6)~~ | ~~10 (5)~~ | | ~~-~~ | | | ~~0.670~~ | | |  |  |  |  |  |  |  |  |
|  | ~~Previous smoking~~ | | | | | | | ~~291 (52)~~ |  | ~~115 (58)~~ | ~~96 (49)~~ | | ~~80 (47)~~ | | | ~~0.062~~ | | |  |  |  |  |  |  |  |  |
|  | ~~Current smoking~~ | | | | | | | ~~81 (14)~~ |  | ~~23 (12)~~ | ~~17 (9)~~ | | ~~41 (24)~~ | | | **~~<0.001~~** | | |  |  |  |  |  |  |  |  |
|  |  | | | ~~Packs/years~~ | | | | ~~0 (0-22)~~ |  | ~~7 (0-30)~~ | ~~0 (0-14)~~ | | ~~0 (0-22)~~ | | | **~~0.008~~** | | |  |  |  |  |  |  |  |  |
|  | ~~Post menopause~~ | | | | | | | ~~249 (44)~~ |  | ~~92 (47)~~ | ~~157 (81)~~ | | ~~-~~ | | | **~~<0.001~~** | | |  |  |  |  |  |  |  |  |
|  | ~~Hormone replacement use~~ | | | | | | | ~~85 (15)~~ |  | ~~16 (8)~~ | ~~69 (35)~~ | | ~~-~~ | | | **~~0.047~~** | | |  |  |  |  |  |  |  |  |
|  | ~~Metabolic syndrome~~ | | | | | | | ~~90 (16)~~ |  | ~~44 (22)~~ | ~~46 (24)~~ | | ~~-~~ | | | ~~0.704~~ | | |  |  |  |  |  |  |  |  |
|  | ~~Aspirin~~ | | | | | |  | ~~102 (18)~~ |  | ~~34 (17)~~ | ~~14 (7)~~ | | ~~54 (32)~~ | | | **~~<0.001~~** | | |  |  |  |  |  |  |  |  |
| ~~Analytical parameters~~ | | | | | | | |  |  |  |  | |  | | |  | | |  |  |  |  |  |  |  |  |
|  | ~~Total cholesterol, mg/dL~~ | | | | | | | ~~197 ± 39~~ |  | ~~195 ± 38~~ | ~~208 ± 37~~ | | ~~186 ± 39~~ | | | **~~<0.001~~** | | |  |  |  |  |  |  |  |  |
|  | ~~Triglycerides, mg/dL~~ | | | | | | | ~~1116 (86-157)~~ |  | ~~-~~ | ~~120 (90-156)~~ | | ~~111 (80-158)~~ | | | ~~0.151~~ | | |  |  |  |  |  |  |  |  |
|  | ~~HDL cholesterol, mg/dL~~ | | | | | | | ~~54 ± 16~~ |  | ~~49 (41-67)~~ | ~~61 ± 15~~ | | ~~43 (37-54)~~ | | | **~~<0.001~~** | | |  |  |  |  |  |  |  |  |
|  | ~~LDL cholesterol, mg/dL~~ | | | | | | | ~~116 ± 33~~ |  | ~~116 ± 31~~ | ~~120 ± 35~~ | | ~~112 ± 33~~ | | | ~~0.100~~ | | |  |  |  |  |  |  |  |  |
|  | ~~Glucose, mg/dL~~ | | | | | | | ~~91 ± 19~~ |  | ~~89 (83-98)~~ | ~~88 (82-94)~~ | | ~~87 (83-94)~~ | | | ~~0.104~~ | | |  |  |  |  |  |  |  |  |
|  | ~~Insulin, uU/ml~~ | | | | | |  | ~~9. 99 ± 7.17~~ |  | ~~5.83 (3.71-9.75)~~ | ~~11.5 (8.60-14.20)~~ | | ~~-~~ | | | **~~<0.001~~** | | |  |  |  |  |  |  |  |  |
|  | ~~Homocysteine,~~ | | | | | | | ~~10.7 ± 3.6~~ |  | ~~9.1 (7.5-10.6)~~ | ~~11.1 (9.6-13.7)~~ | | ~~10.5 ± 3.4~~ | | | **~~<0.001~~** | | |  |  |  |  |  |  |  |  |
|  | ~~Creatinine, mg/dL~~ | | | | | | | ~~0.8 ± 0.3~~ |  | ~~0.8 ± 0.2~~ | ~~0.9 ± 0.3~~ | | ~~0.8 ± 0.2~~ | | | **~~0.014~~** | | |  |  |  |  |  |  |  |  |
|  | ~~CRP, mg/L~~ | | | | | |  | ~~4.65 (1.75-12.50)~~ |  | ~~-~~ | ~~5.62 (1.97-13.35)~~ | | ~~4.00 (1.22-13.02)~~ | | | ~~0.035~~ | | |  |  |  |  |  |  |  |  |
| ~~RA associated characteristics~~ | | | | | | | |  |  |  |  | |  | | |  | | |  |  |  |  |  |  |  |  |
|  | ~~Disease duration, years~~ | | | | | | | ~~10 (3-20)~~ |  | ~~9 (4-17)~~ | ~~13 (7-23)~~ | | ~~3 (2-18)~~ | | | **~~<0.001~~** | | |  |  |  |  |  |  |  |  |
|  | ~~Prednisone~~ | | | | | |  |  |  |  |  | |  | | |  | | |  |  |  |  |  |  |  |  |
|  |  | | ~~Ever prednisone treatment~~ | | | | | ~~458 (82)~~ |  | ~~147 (75)~~ | ~~172 (88)~~ | | ~~139 (82)~~ | | | **~~0.002~~** | | |  |  |  |  |  |  |  |  |
|  |  | | ~~Current treatment with prednisone~~ | | | | | ~~246 (44)~~ |  | ~~76 (39)~~ | ~~78 (40)~~ | | ~~92 (54)~~ | | | **~~<0.001~~** | | |  |  |  |  |  |  |  |  |
|  |  | | ~~Current prednisone dosage, mg/day~~ | | | | | ~~0 (0-5)~~ |  | ~~0 (0-5)~~ | ~~0 (0-5)~~ | | ~~2 (0-5)~~ | | | **~~0.011~~** | | |  |  |  |  |  |  |  |  |
|  | ~~Hydroxychloroquine~~ | | | | | | | ~~125 (22)~~ |  | ~~47 (24)~~ | ~~36 (18)~~ | | ~~42 (25)~~ | | | ~~0.277~~ | | |  |  |  |  |  |  |  |  |
|  | ~~Methotrexate~~ | | | | | | | ~~360 (64)~~ |  | ~~125 (63)~~ | ~~115 (59)~~ | | ~~120 (71)~~ | | | ~~0.056~~ | | |  |  |  |  |  |  |  |  |
|  | ~~TNF inhibitors~~ | | | | | | | ~~179 (32)~~ |  | ~~85 (43)~~ | ~~59 (30)~~ | | ~~35 (21)~~ | | | **~~<0.001~~** | | |  |  |  |  |  |  |  |  |
|  | ~~Anakinra~~ | | | | | |  | ~~2 (0)~~ |  | ~~1 (1)~~ | ~~1 (1)~~ | | ~~0 (0)~~ | | | ~~0.649~~ | | |  |  |  |  |  |  |  |  |
|  | ~~NSAIDs~~ | | | | | |  | ~~313 (73)~~ |  | ~~127 (64)~~ | ~~135 (69)~~ | | ~~51 (30)~~ | | | **~~<0.001~~** | | |  |  |  |  |  |  |  |  |
|  | ~~COX2 inhibitors~~ | | | | | | | ~~154 (72)~~ |  | ~~47 (24)~~ | ~~56 (29)~~ | | ~~51 (30)~~ | | | ~~0.356~~ | | |  |  |  |  |  |  |  |  |
|  | ~~Minutes of morning stiffness~~ | | | | | | | ~~30 (5-60)~~ |  | ~~15 (5-30)~~ | ~~30 (0-60)~~ | | ~~30 (10-90)~~ | | | **~~<0.001~~** | | |  |  |  |  |  |  |  |  |
|  | ~~Current biologic DMARD use~~ | | | | | | | ~~149 (27)~~ |  | ~~89 (45)~~ | ~~60 (31)~~ | | ~~-~~ | | | **~~0.003~~** | | |  |  |  |  |  |  |  |  |
|  | ~~Any current use of non-biologic DMARDs~~ | | | | | | | ~~487 (87)~~ |  | ~~165 (84)~~ | ~~175 (90)~~ | | ~~147 (87)~~ | | | ~~0.265~~ | | |  |  |  |  |  |  |  |  |
|  | ~~Joint surgery~~ | | | | | | | ~~156 (28)~~ |  | ~~55 (28)~~ | ~~101 (52)~~ | | ~~-~~ | | | **~~<0.001~~** | | |  |  |  |  |  |  |  |  |
|  | ~~Rheumatoid nodules~~ | | | | | | | ~~197 (35)~~ |  | ~~89 (45)~~ | ~~108 (55)~~ | | ~~-~~ | | | **~~0.012~~** | | |  |  |  |  |  |  |  |  |
|  | ~~Global assessment of disease activity~~ | | | | | | | ~~24 (9-47)~~ |  | ~~21 (5-47)~~ | ~~19 (6-34)~~ | | ~~30 (16-55)~~ | | | **~~<0.001~~** | | |  |  |  |  |  |  |  |  |
|  | ~~Modified HAQ (VU cohort only)~~ | | | | | | | ~~0.500 (0.000-0.875)~~ |  | ~~-~~ | ~~-~~ | | ~~0.500 (0.000-0.875)~~ | | | ~~-~~ | | |  |  |  |  |  |  |  |  |
|  | ~~DAS28 (ESCAPE and VU cohorts)~~ | | | | | | | ~~3.72 ± 1.35~~ |  | ~~3.66 ± 1.08~~ | ~~-~~ | | ~~3.79 ± 1.61~~ | | | ~~0.362~~ | | |  |  |  |  |  |  |  |  |
|  | ~~Full HAQ (ESCAPE cohort only)~~ | | | | | | | ~~0.625 (0.125-1.250)~~ |  | ~~0.625 (0.125-1.250)~~ | ~~-~~ | | ~~-~~ | | | ~~-~~ | | |  |  |  |  |  |  |  |  |
|  | ~~RF (>40 units)~~ | | | | | | | ~~363 (65)~~ |  | ~~129 (65)~~ | ~~117 (60)~~ | | ~~117 (69)~~ | | | ~~0.136~~ | | |  |  |  |  |  |  |  |  |
| ~~Data expressed as mean (± standard deviation) or median (interquartile range). Dichotomous variables are expressed as number (percentage).~~ | | | | | | | | | | | | | | | | | |  | | | | | |  | | |
| ~~CRP=C reactive protein; HAQ=Health Assessment Questionnaire; DAS28=Disease Activity Score.~~  ~~HDL=High density lipoprotein; LDL=Low density lipoprotein.~~ | | | | | | | | | | | | | | | | | | | | | |  | | | |  |
| ~~DMARD=Disease-modifying Antirheumatic Drug; TNF=tumor necrosis factor; COX-2=cyclooxygenase-2~~ | | | | | | | | | | | |  | |  | | | | | |  | | |  |  |  |  |
| ~~Modified HAQ is only available for Vanderbilt cohort. DAS28 only for ESCAPE and Vanderbilt cohort. Full HAQ only in ESCAPE series.~~  ~~Significant p value are depicted in bold. Comparisons are performed through Chi2, ANOVA or Kruskall-Wallis method.~~ | | | | | | | | | | | | | | |  | | | | | |  | | | |  |  |

| **Supplementary Table 4. Univariable association of demographic, co-morbidity and RA related data with CAC** | | | | | | | | | |  |  |
| --- | --- | --- | --- | --- | --- | --- | --- | --- | --- | --- | --- |
|  |  | | |  |  | OR (95% CI), p | | | |  |  |
|  |  | | |  |  | CAC > 100 | | CAC > 300 | |  |  |
| Demographics | | | | |  |  |  |  |  |  |  |
|  | Age, years | | | | | 1.11 (1.08-1.13) | **<0.001** | 1.12 (1.08-1.15) | **<0.001** |  |  |
|  | Male | | | |  | 3.56 (2.35-5.38) | **<0.001** | 3.73 (2.32-5.98) | **<0.001** |  |  |
|  | BMI, kg/m2 | | | | | 0.99 (0.96-1.02) | 0.55 | 0.99 (0.95-1.03) | 0.61 |  |  |
|  | Hip circumference, cm | | | | | 0.99 (0.98-1.01) | 0.25 | 0.99 (0.98-1.01) | 0.43 |  |  |
|  | Waist circumference, cm | | | | | 1.02 (1.01-1.03) | **0.004** | 1.02 (1.00-1.03) | **0.021** |  |  |
| Comorbidity factors | | | | | |  |  |  |  |  |  |
|  | Hypertension, n(%) | | | | | 3.08 (2.10-4.51) | **<0.001** | 2.54 (1.58-4.09) | **<0.001** |  |  |
|  | Systolic blood pressure, mmHg | | | | | 1.02 (1.01-1.03) | **<0.001** | 1.03 (1.02-1.04) | **<0.001** |  |  |
|  | Diastolic blood pressure, mmHg | | | | | 1.01 (0.99-1.03) | 0.178 | 1.01 (0.99-1.04) | 0.21 |  |  |
|  | Use of antihypertensives, n(%) | | | | | 2.98 (2.05-4.33) | **<0.001** | 2.42 (1.53-3.80) | **<0.001** |  |  |
|  | Dyslipidemia | | | | | 1.09 (0.86-1.37) | 0.47 | 1.08 (0.83-1.41) | 0.56 |  |  |
|  |  | | On lipid lowering drugs | | | 3.73 (2.11-6.60) | **<0.001** | 3.84 (2.04-7.24) | **<0.001** |  |  |
|  | Diabetes | | | |  | 4.28 (2.10-8.73) | **<0.001** | 2.84 (1.10-7.29) | **0.030** |  |  |
|  | Previous smoking | | | | | 2.96 (2.01-4.35) | **<0.001** | 3.36 (2.02-5.57) | **<0.001** |  |  |
|  | Current smoking | | | | | 1.11 (0.67-1.85) | 0.68 | 0.87 (0.45-1.68) | 0.68 |  |  |
|  |  | | Packs/years | | | 1.02 (1.01-1.02) | **<0.001** | 1.02 (1.01-1.03) | **0.001** |  |  |
|  | Post menopause | | | | | 0.74 (0.48-1.16) | 0.19 | 0.68 (0.39-1.18) | 0.17 |  |  |
|  | Hormone replacement use | | | | | 1.27 (0.70-2.29) | 0.43 | 0.95 (0.41-2.19) | 0.91 |  |  |
|  | Metabolic syndrome | | | | | 1.38 (0.83-2.27) | 0.21 | 1.28 (0.68-2.40) | 0.45 |  |  |
|  | Aspirin | | | |  | 2.98 (1.91-4.63) | **<0.001** | 3.18 (1.94-5.24) | **<0.001** |  |  |
| Analytical parameters | | | | | |  |  |  |  |  |  |
|  | Total cholesterol, mg/dL | | | | | 1.00 (0.99-1.00) | 0.20 | 1.00 (0.99-1.00) | 0.57 |  |  |
|  | Triglycerides, mg/dL | | | | | 1.00 (0.99-1.00) | 0.75 | 1.00 (1.00-1.00) | 0.57 |  |  |
|  | HDL cholesterol, mg/dL | | | | | 0.98 (0.97-0.99) | **0.003** | 0.98 (0.97-0.99) | **0.014** |  |  |
|  | LDL cholesterol, mg/dL | | | | | 1.00 (0.99-1.01) | 0.92 | 1.00 (0.99-1.01) | 0.57 |  |  |
|  | Glucose, mg/dL | | | | | 1.02 (1.01-1.03) | **0.001** | 1.01 (1.00-1.03) | **0.008** |  |  |
|  | Insulin, | | | |  | 1.01 (0.98-1.04) | 0.39 | 0.99 (0.95-1.04) | 0.77 |  |  |
|  | Homocysteine, | | | | | 1.07 (1.02-1.13) | **0.006** | 1.12 (1.06-1.18) | **<0.001** |  |  |
|  | Creatinine, mg/dL | | | | | 4.02 (1.85-8.75) | **<0.001** | 6.61 (2.65-16.49) | **<0.001** |  |  |
|  | CRP, mg/L | | | | | 1.01 (0.99-1.03) | 0.091 | 1.01 (0.99-1.03) | 0.24 |  |  |
| Rheumatoid arthritis related data | | | | | |  |  |  |  |  |  |
|  | Disease duration, years | | | | | 1.03 (1.01-1.05) | **<0.001** | 1.02 (1.00-1.04) | **0.026** |  |  |
|  | Prednisone | | | | |  |  |  |  |  |  |
|  |  | Ever prednisone treatment | | | | 1.03 (0.64-1.66) | 0.89 | 1.28 (0.69-2.36) | 0.44 |  |  |
|  |  | Current with prednisone | | | | 1.00 (0.69-1.43) | 0.98 | 0.88 (0.56-1.39) | 0.59 |  |  |
|  |  | Current prednisone dosage, mg/day | | | | 1.00 (0.96-1.05) | 0.96 | 0.98 (0.92-1.04) | 0.45 |  |  |
|  | Hydroxychloroquine | | | | | 0.80 (0.51-1.24) | 0.32 | 0.58 (0.32-1.07) | 0.080 |  |  |
|  | Methotrexate | | | | | 0.64 (0.44-0.93) | **0.018** | 0.55 (0.35-0.87) | **<0.001** |  |  |
|  | TNF inhibitors | | | | | 1.04 (0.70-1.53) | 0.86 | 0.92 (0.56-1.49) | 0.72 |  |  |
|  | NSAIDs | | | |  | 0.85 (0.59-1.22) | 0.37 | 0.54 (0.35-0.85) | **0.008** |  |  |
|  | COX2 inhibitors | | | | | 0.98 (0.66-1.48) | 0.94 | 0.75 (0.44-1.27) | 0.28 |  |  |
|  | Current biologic DMARD use | | | | | 1.06 (0.68-1.66) | 0.79 | 1.06 (0.68-1.66) | 0.79 |  |  |
|  | Any current use of non-biologic DMARDs | | | | | 0.65 (0.39-1.09) | 0.10 | 0.80 (0.43-1.51) | 0.49 |  |  |
|  | Joint surgery | | | | | 0.99 (0.64-1.54) | 0.96 | 1.15 (0.66-2.02) | 0.62 |  |  |
|  | Rheumatoid nodules | | | | | 1.02 (0.66-1.58) | 0.94 | 1.15 (0.65-2.02) | 0.63 |  |  |
|  | Global assessment of disease activity | | | | | 1.00 (0.99-1.01) | 0.50 | 1.00 (0.99-1.01) | 0.65 |  |  |
|  | Modified HAQ (VU cohort only) | | | | | 1.79 (0.92-3.47) | 0.09 | 1.68 (0.80-3.55) | 0.17 |  |  |
|  | DAS28 (ESCAPE and VU cohorts) | | | | | 1.12 (0.95-1.32) | 0.16 | 1.10 (0.90-1.33) | 0.37 |  |  |
|  | Full HAQ (ESCAPE cohort only) | | | | | 1.08 (0.72-1.60) | 0.72 | 1.02 (0.62-1.67) | 0.95 |  |  |
|  | RF (>40 units) | | | | | 1.33 (0.90-1.98) | 0.16 | 1.31 (0.80-2.15) | 0.29 |  |  |
| Data expressed as mean (± standard deviation) or median (interquartile range).  Dichotomous variables are expressed as number (percentage). | | | | | | | | | | | |
| CRP=C reactive protein; HAQ=Health Assessment Questionnaire; DAS28=Disease Activity Score;  HDL=High density lipoprotein; LDL=Low density lipoprotein; | | | | | | | | | | | |
| DMARD=Disease-modifying Antirheumatic Drug; TNF=tumor necrosis factor; COX-2=cyclooxygenase-2; | | | | | | | | | | |  |
| CAC -coronary artery calcification- percentiles were adjusted for age and race as previously described.  Significant p value are depicted in bold. | | | | | | | | | | |  |
| Modified HAQ is only available for Vanderbilt cohort. DAS28 only for ESCAPE and Vanderbilt cohort.  Full HAQ only in ESCAPE series. | | | | | | | | | | | |

| **~~Supplementary Table 6. Internal validation of the predictive model containing ACCA/AHA score, RA related data and cytokines~~** | | | | | |
| --- | --- | --- | --- | --- | --- |
|  | ~~CAC > 100~~ | ~~CAC > 300~~ |  |  |  |
| ~~Groups~~ |  |  |  |  |  |
| ~~k1~~ | ~~0.142~~ | ~~0.123~~ |  |  |  |
| ~~k2~~ | ~~0.323~~ | ~~0.186~~ |  |  |  |
| ~~k3~~ | ~~0.181~~ | ~~0.104~~ |  |  |  |
| ~~k4~~ | ~~0.303~~ | ~~0.183~~ |  |  |  |
| ~~k5~~ | ~~0.177~~ | ~~0.392~~ |  |  |  |
| ~~pseudo R2 mean~~ | ~~0.225~~ | ~~0.223~~ |  |  |  |
| ~~ACCA/AHA score: American College of Cardiology and the American Heart Association CV risk score.~~ | | | |  |  |
| ~~RA: rheumatoid arthritis.~~ | |  |  |  |  |
| ~~CAC: coronary artery calcification.~~ | |  |  |  |  |
